# Benefits of Real-Time Continuous Glucose Monitoring in Pregnancy

**DOI:** 10.1089/dia.2020.0667

**Published:** 2021-03-02

**Authors:** Jennifer M. Yamamoto, Helen R. Murphy

**Affiliations:** ^1^ Department of Internal Medicine, College of Medicine, Rady Faculty of Health Sciences, University of Manitoba, Winnipeg, Canada.; ^2^ Department of Medicine, Cumming School of Medicine, University of Calgary, Calgary, Canada.; ^3^ Cambridge University Hospitals NHS Foundation Trust, Cambridge, UK.; ^4^ Division of Women's and Children's Health, Women's Health Academic Centre, King's College London, London, UK.; ^5^ Norwich Medical School, University of East Anglia, Norwich, UK.

**Keywords:** Pregnancy, Diabetes, Continuous glucose monitoring, Technology, Gestational diabetes, Type 1 diabetes

## Abstract

In recent years, continuous glucose monitoring (CGM) has become increasingly available with the introduction of devices that are specifically approved for use during pregnancy. Evidence in the form of randomized-controlled trials and cohort studies continues to build support for the use of CGM during pregnancy to improve measures of maternal glycemia as well as obstetric and neonatal outcomes. Based on data from the CGM in pregnant women with type 1 diabetes (CONCEPTT) trial alongside a Swedish cohort study of real-world outcomes of pregnant women with type 1 diabetes, the UK National Institute for Health and Clinical Excellence (NICE) guidelines now recommend that real-time CGM be offered to all pregnant women with type 1 diabetes. Based on these guidelines, all pregnant individuals in the United Kingdom with type 1 diabetes will receive government-funded real-time CGM for a 12-month duration. These guidelines are a game-changer and will continue to facilitate more widespread access to CGM use in the United Kingdom and beyond. This review describes the role of CGM in the management of diabetes in pregnancy, discusses contemporary maternal glucose levels and their relationship with outcomes in diabetes pregnancies, and examines the high-quality, randomized-controlled trial and the real-world clinical data evaluating the impact of CGM use.

## Introduction

While continuous glucose monitoring (CGM) has been available for the past two decades, it is only in recent years that we have had access to devices that are specifically approved for use during pregnancy. Undoubtedly, the increasing availability, affordability, and usability of the FreeStyle Libre (also known as intermittent CGM or flash), which is CE marked for use in pregnancy, have transformed the clinical management of diabetes before and during pregnancy.^1^ The Dexcom G6 is a game changer in terms of real-time CGM with hypoglycemia alerts and alarms. It is accurate enough to replace self-monitoring of blood glucose (SMBG) for precise prandial insulin dosing in type 1 diabetes pregnancy and is also CE marked for use during pregnancies complicated by type 1 diabetes, type 2 diabetes, and gestational diabetes.^2^ The Dexcom G6 further benefits from its interoperability, meaning that it can be used either as a standalone real-time CGM system for those using insulin pumps or multiple daily injections or together with a subcutaneous insulin pump (currently a DANA pump in Europe or Tandem T-slim pump in North America) as part of an automated insulin delivery system. As evidence supporting the use of CGM in pregnancy continues to build, it is important to understand how CGM can be leveraged to improve pregnancy outcomes as well as to better characterize the relationship between maternal glycemia and diabetes-related complications in pregnancy. The aim of this review is to describe contemporary maternal glucose levels and their relationship with obstetric and neonatal outcomes in diabetes pregnancies and to examine the high-quality, randomized-controlled trial and the real-world clinical data evaluating the impact of CGM use.

## The Changing Landscape of Diabetes in Pregnancy

The prevalence of gestational diabetes and of pregnancies complicated by type 1 or type 2 diabetes has doubled over recent decades, such that by the age of 30 years, almost 1 in 10 women is affected by hyperglycemia in pregnancy.^3^ The increasing prevalence of pregnancies complicated by hyperglycemia is apparent across all types of diabetes, affecting not only women with gestational and type 2 diabetes, but also women with type 1 diabetes, in whom increases of 30%–40% have been reported across Northern Europe.^4^ It is well established that pregnant women with type 1 diabetes have higher and more variable glucose levels, are more likely to have impaired awareness to hypoglycemia, increased day to day hypoglycemic events, and increased risk of severe hypoglycemia, and thus spend less time with glucose levels in the pregnancy target range compared with pregnant women with type 2 diabetes. For women with type 1 diabetes, who lack biologically active pancreatic insulin secretion, it is particularly challenging to balance the achievement of tight pregnancy glucose targets with the risk of daily and severe hypoglycemic events. Consequently, only a minority (∼15%) are able to achieve the recommended hemoglobin A1C (HbA1c) target of below 48 mmol/mol (6.5%) in early pregnancy.^5^

Nationwide population-based data from 8685 pregnant women with type 1 diabetes demonstrate that younger aged women (younger than 25 years), those with more than a 5-year duration of diabetes, higher body mass index (BMI >25 kg/m^2^), and living in all except for the least deprived regions are unlikely to achieve glycemic targets.^6^ Indeed, the women most likely to achieve target HbA1c levels using SMBG are those aged 35–44 years (who generally have higher incomes, education and health literacy) and those with the shortest duration of diabetes (<1 year) who likely have residual pancreatic insulin secretion (Table 1).^6^

**Table 1.** Factors Associated with Target Glycemia (Hemoglobin A1C Less than 6.5% [48 mmol/mol]) in Early Pregnancy

Type 1 diabetes pregnancy		Type 2 diabetes pregnancy	
Category	Odds ratio (95% CI)	Category	Odds ratio (95% CI)
Maternal age category^a^		Ethnicity^e^	
Age 15–24 years	0.32 (0.25–0.40)	Ethnicity N/A	0.99 (0.79–1.24)
Age 35–44 years	1.30 (1.11–1.52)	Unknown	1.79 (1.16–2.76)
Age >45 years	2.14 (0.65–7.13)	Mixed	1.10 (0.76–1.58)
		Asian	0.72 (0.64–0.82)
		Black	0.72 (0.59–0.88)
		Other	0.86 (0.61–1.20)
Maternal deprivation quintile^b^			
Deprivation N/A	0.70 (0.51–0.96)	Deprivation N/A	0.99 (0.73–1.34)
Quintile 5	0.44 (0.35–0.55)	Quintile 5	0.73 (0.57–0.93)
Quintile 4	0.48 (0.39–0.60)	Quintile 4	0.84 (0.65–1.07)
Quintile 3	0.65 (0.53–0.80)	Quintile 3	1.01 (0.78–1.30)
Quintile 2	0.67 (0.54–0.82)	Quintile 2	1.09 (0.82–1.45)
Diabetes duration			
Diabetes duration N/A^c^	0.76 (0.38–1.51)	Diabetes duration N/A	0.79 (0.59–1.06)
Duration <1 year	1.69 (1.11–2.57)	Duration <1 year	0.86 (0.72–1.02)
Duration 5–9 years	0.54 (0.43–0.67)	Duration 5–9 years	0.60 (0.52–0.68)
Duration 10–14 years	0.40 (0.32–0.50)	Duration 10–14 years	0.48 (0.39–0.60)
Duration >15 years	0.43 (0.36–0.52)	Duration >15 years	0.41 (0.27–0.61)
BMI (kg/m^2^)			
BMI <18.5^d^	1.27 (0.71–2.29)	BMI <18.5	0.86 (0.36–2.10)
BMI 25–29.9	0.75 (0.64–0.87)	BMI 25–29.9	0.80 (0.66–0.97)
BMI 30–34.9	0.63 (0.51–0.77)	BMI 30–34.9	0.65 (0.54–0.79)
BMI 35–39.9	0.55 (0.39–0.78)	BMI 35–39.9	0.55 (0.45–0.68)
BMI >40	0.53 (0.31–0.90)	BMI >40	0.52 (0.42–0.64)

Separate models were run for type 1 and type 2 diabetes. Only factors that were significant in univariate analyses were retained in the multivariate analyses. N/A is data not available.

^a^ Reference group for maternal age category at delivery is 25–34 years. Maternal age category did not improve the model fit in type 2 diabetes and is therefore omitted.

^b^ Reference group for maternal deprivation quintile is the least deprived quintile 1.

^c^ Reference group for diabetes duration is duration 1–4 years.

^d^ Reference group for maternal BMI is the healthy BMI range of 18.5–24.9.

^e^ Reference group for maternal ethnicity is white.

BMI, body mass index.

In contrast to previous data suggesting substantial clinic to clinic variation, this large cohort demonstrated that after adjusting for maternal demographic and clinical characteristics, there was no evidence of statistically significant variation in maternal glycemia between clinics, either in early or late gestation.^5,6^ In addition, the already high rates of preterm delivery (before 37 weeks) and of large-for-gestational-age birthweight in type 1 diabetes pregnancy also seem to be increasing in recent years and these patterns are also apparent across all diabetes pregnancy clinics and without substantial clinic to clinic variation.^6^ Thus, new interventions to safely optimize antenatal glucose levels and reduce obstetric and neonatal complications are needed, for all pregnant women with type 1 diabetes, and these should be implemented across all maternity clinics.

## Growing Evidence Supporting CGM Use in Pregnancy

The CONCEPTT (CGM in pregnant women with type 1 diabetes) trial provided high-quality, randomized-controlled trial data demonstrating that the use of real-time CGM was associated with lower HbA1c at 34 weeks, suggesting improved maternal glucose levels during the late second and early third trimesters.^7^ Importantly, this was accompanied by 7% higher time in range (TIR) and 5% lower time above range (TAR) without increasing maternal hypoglycemia. Beyond impacting surrogate markers of maternal glycemia, using CGM led to clinically significant reductions in large for gestational-age infants, neonatal hypoglycemia, and neonatal intensive care unit (NICU) admissions.^7^ A systematic review combining data from CONCEPTT with that of the type 1 diabetes arm of the GlucoMOMS trial also showed evidence for a reduction in preeclampsia.^7–9^ This is important because preeclampsia and its consequences of growth restriction and preterm births are associated with substantial mother and infant morbidity and mortality globally. Mother and infant deaths account for 6%–7% of all deaths globally, approximately half of the global burden of deaths caused by cancer.^10^

A Technology Appraisal from National Health Service (NHS) Wales reported that CGM use improves glycemic control, reduces the incidence of preeclampsia and neonatal hypoglycemia, and reduces admission to and duration of stay in neonatal intensive care.^8^ All NHS Trusts must now adopt this guidance, and if not, they are required to justify why it has not been followed.^11^ Based on the data from CONCEPTT and the real-world health outcomes of pregnant women with type 1 diabetes and their newborns in the Swedish study, the UK National Institute for Health and Clinical Excellence (NICE) have also reviewed their diabetes pregnancy guidelines (Table 2).

**Table 2.** Summary of National Institute for Health and Clinical Excellence Guideline Recommendations for Use of Continuous Glucose Monitoring in Pregnancy

Offer CGM to all pregnant women with type 1 diabetes to help them meet their pregnancy blood glucose targets and improve neonatal outcomes.
Offer intermittently scanned CGM (commonly referred to as flash) to pregnant women with type 1 diabetes who are unable to use CGM or express a clear preference for it.
Consider CGM for pregnant women who are on insulin therapy but do not have type 1 diabetes, if: They have problematic severe hypoglycemia (with or without impaired awareness of hypoglycemia) or They have unstable blood glucose levels that are causing concern despite efforts to optimize glycemic control.
For pregnant women who are using intermittently scanned CGM or CGM, a member of the joint diabetes and antenatal care team with expertise in these systems should provide education and support (including advising women about sources of out-of-hours support).

CGM, continuous glucose monitoring; NICE, National Institute for Health and Clinical Excellence.

The NICE guidelines were updated in December 2020 to recommend that real-time CGM be offered to all pregnant women with type 1 diabetes.^12^ All pregnant women with type 1 diabetes will receive government-funded real-time CGM for up to 12 months of duration, meaning that they can continue CGM use beyond pregnancy into the early postnatal period. They also advise that a member of the clinical team with expertise in CGM and flash should provide education and support, including advising women about sources of out-of-hours support. A list of web resources for providing out-of-hours support is provided. This is a potential game changer for pregnant women with type 1 diabetes and health care professionals, in the United Kingdom and beyond.

In addition to randomized-controlled trial evidence, real-world data on the use of real-time CGM and flash glucose monitoring in type 1 diabetes pregnancies have confirmed the association between CGM measures and neonatal outcomes.^13^ A Swedish study of 186 women described the use of real-time CGM (Dexcom G4) and flash glucose monitoring (FreeStyle Libre) in type 1 diabetes pregnancies. Authors demonstrated that a higher CGM TIR in the second and third trimesters was associated with a decreased risk of large-for-gestational-age and a decreased risk of their composite neonatal outcome (shoulder dystocia, macrosomia, neonatal hypoglycemia, and neonatal intensive care admission). When taken together, these randomized-controlled trial and real-world data suggest that increasing TIR by 5% is associated with clinically meaningful improvements in outcomes.^7,13^

Data regarding the use of CGM in pregnant women with type 2 diabetes are still limited. Three randomized trials performed in the United Kingdom, Denmark, and the Netherlands included pregnant women with both type 1 and type 2 diabetes.^9,14,15^ However, only small numbers of pregnant women with type 2 diabetes were included (35%, 20%, and 27% for the United Kingdom, Denmark, and the Netherlands, respectively).^9,14,15^ Furthermore, the UK and Dutch studies used older generation masked CGM sensors (known as professional or retrospective CGM) and the Danish study used real-time CGM intermittently rather than continuously throughout pregnancy.^14,15^

Only the UK study published details of the gestational changes in CGM glucose profiles during type 2 diabetes pregnancy showing ∼60% TIR during the first trimester, rising to 65% in the second and 75% in the third trimester.^16^ In contrast to women with type 1 diabetes, who had only modest reductions in TAR, women with type 2 diabetes had substantial gestational improvements in TAR from ∼33% (8 h/day) in trimester 1, 20% (4.8 h/day) in trimester 2, and 12% (3 h/day) in trimester 3.^16^ The reduced fetal exposure to maternal hyperglycemia likely explains the significantly lower rates of preterm births, large-for-gestational-age birthweight infants, and neonatal intensive care unit admissions in type 2 diabetes offspring.

**FIG. 1.**
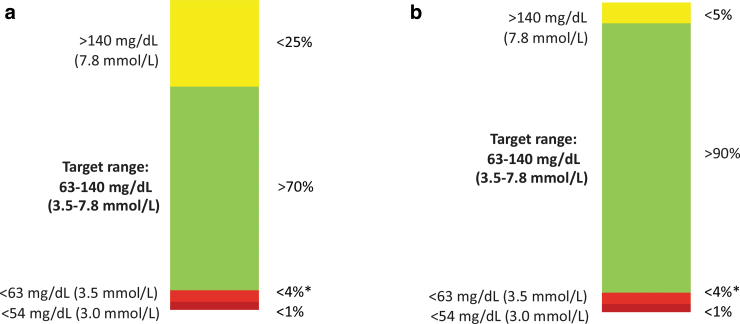
Continuous glucose monitoring targets in pregnancy (Adapted from Battelino et al.^18^) for **(a)** type 1 diabetes and **(b)** type 2 diabetes. *Includes percentage of values <3.0 mmol/L.

While the achievement of glucose targets in pregnancy is more common in women with type 2 diabetes compared with those with type 1 diabetes, it would be a mistake to assume that type 2 diabetes is a milder or less serious concern in pregnancy. It is possible that tighter glucose targets may be needed for optimal pregnancy outcomes in type 2 diabetes.^17^ The recent international consensus for CGM-derived glycemic targets suggested that 90% TIR 3.5–7.8 mmol/L (63–140 mg/dL) may be applicable with no more than 5% TAR, and 4% time below range (TBR) may be applicable during type 2 diabetes pregnancy (Fig. 1).^18^ However, given the paucity of CGM data and no studies describing associations with pregnancy outcome, there are no evidence-based recommendations in type 2 diabetes pregnancy.^18^ Given the global increase in type 2 diabetes pregnancy and widespread availability of more affordable user-friendly CGM devices, data regarding the pregnancy outcomes and costs and benefits of CGM use in this marginalized patient population are urgently needed.

While CGM may also have potential for pregnancies in individuals with gestational diabetes, evidence supporting the use of real-time CGM remains limited. Randomized-controlled trial evidence in gestational diabetes has been limited to the use of retrospective or intermittent CGM.^9,19^ Additional trials are needed to determine if women with gestational diabetes would benefit from real-time CGM and/or flash glucose monitoring.

## Using CGM and Closed-Loop Studies to Understand How to Achieve Glucose Targets

The CONCEPTT and Swedish studies of CGM in pregnancy demonstrated the gap that currently exists between the CGM TIR targets and the realities of safely achieving 70% TIR with no more than 25% TAR in type 1 diabetes pregnancy.^7,13^ In both studies, women using multiple daily injections or insulin pump therapy and with access to flash or CGM only achieved the pregnancy glucose targets during the third trimester. While there is an association between CGM TIR and fetal complications both at 24 and at 34 weeks, earlier achievement of the pregnancy TIR glucose levels is needed for optimal neonatal outcomes.^7,13,20^ It is clear that the use of CGM alone is not adequate in achieving glycemic targets and reducing complications in pregnancy.

Closed-loop insulin delivery may help to further close the gap between current and optimal pregnancy outcomes. Thus far data regarding the safety and efficacy of closed-loop insulin delivery in type 1 diabetes are limited. Early feasibility studies conducted under strict experimental laboratory conditions suggested that closed-loop systems could safely escalate overnight basal insulin delivery as pregnancy advances and that CGM sensor accuracy was acceptable both in early and in late gestation.^21^ In addition to early feasibility studies, two small crossover randomized-controlled trials examining overnight closed-loop insulin delivery and day-and-night closed-loop insulin delivery have been performed.^22,23^ The overnight closed-loop randomized crossover trial demonstrated an increase in overnight TIR of 15% compared with sensor-augmented pump therapy.^22^ While the crossover trial of day-and-night closed-loop insulin delivery did not demonstrate a significant increase in TIR in a more diverse group of participants with higher baseline HbA1c, it did show a significant decrease in TBR and significantly fewer episodes of hypoglycemia.^23^

While early closed-loop insulin delivery studies are promising for future clinical care, they also give us additional insight into the importance of optimizing diet and exercise in helping women to achieve the tight pregnancy glucose targets. Stable-label isotope studies demonstrated the relatively small contribution of decreasing hepatic insulin sensitivity compared with the very substantial increase in peripheral insulin resistance, which is essential for postmeal glucose uptake.^24^ Indeed, postmeal glucose disposal is substantially delayed in late pregnancy, with weekly delays from 12 to 16 weeks onward. This is further exacerbated by delayed insulin absorption with the time from peak plasma insulin concentration increasing from ∼50 min in early pregnancy to 80 min in late pregnancy.^24^

The slower achievement of maximal postprandial plasma insulin concentration and increased peripheral insulin resistance, together impede postprandial glucose uptake, facilitating the prolonged duration of postprandial hyperglycemia in late pregnancy. Thus, in addition to aggressively increasing premeal boluses to stimulate peripheral muscle glucose uptake, it is also important to administer earlier premeal boluses as pregnancy advances. This is as essential for women using closed-loop as it is in routine clinical care. However, it is further complicated by the fact that unlike outside of pregnancy where insulin absorption varies greatly between individuals, during pregnancy there is marked intraindividual variations such that pregnant women can administer the same bolus dose with different absorption profiles from one day to the next.^25^

An interesting observation from the day and night closed-loop feasibility studies was that even with insulin pumps and CGM, most women struggled to achieve glucose targets in real life but were able to do so under experimental laboratory conditions with supervised physical activity and dietary intake.^21^ Women were provided with a choice of standardized meals (breakfast 50 g and dinner 60 g of carbohydrate) and 15–30 g between-meal carbohydrate snacks. They performed a morning (09:30 h) and afternoon (15:30 h) supervised treadmill exercise session (50 min of treadmill walking divided into 2 × 25-min sessions) in addition to short postprandial walks after breakfast, lunch, and dinner. This had a striking impact on maternal glucose levels with and without closed-loop insulin delivery during the second trimester. Specifically, with meticulous attention to diet and physical activity, women (aged 32.9 years, duration of type 1 diabetes 17.6 years, BMI 27.1 kg/m^2^) achieved 80% TIR at ∼20–24 weeks of gestation, increasing to 95%–100% during the overnight period. However, the impact of closed-loop was most apparent during the overnight period with a 15% higher TIR and strikingly less intraindividual variability (98 [94–100] vs. 83 [50–100]) compared with CGM and insulin pump therapy.^21^

Closed-loop insulin delivery was also potentially safer in terms of hypoglycemia reduction, with fewer hypoglycemia events and significantly less time below 2.5 mmol/L (45 mg/dL). Closed-loop could not prevent exercise-related hypoglycemia, and so, fast-acting carbohydrates were still required especially for postmeal exercise when prandial insulin is still active. These data, using older generation CGM sensors, also suggested that CGM users should consider additional SMBG during and after exercise.^26^ Although the impact of exercise using current-generation sensors is not clear, using SMBG to conform and monitor treatment of hypoglycemia remains prudent.

When the glycemic profiles and physical activity energy expenditure patterns were compared with what women did in their everyday lives, the overall physical activity energy expenditure did not substantially differ, but the 24-h mean glucose concentration was strikingly lower (7.7 vs. 6.0 mmol/L) with structured diet and exercise. Furthermore, while most women achieved the recommended 30 min of daily exercise, the lack of activity from 7 pm onward, usually after the largest daily carbohydrate intake, was particularly striking and accompanied by almost 20% higher overnight TAR.^27^ The data suggest that carefully controlled carbohydrate intake and structured daily exercise may be needed to overcome the increased peripheral insulin resistance and limit the duration of postprandial hyperglycemia during type 1 diabetes pregnancy.

Recent insights into maternal dietary intake were obtained from some of the CONCEPTT secondary analyses.^28,29^ The application of functional data analysis to CGM data illustrates specific variations in temporal glucose profiles across the 24-h day, which are not captured by TIR metrics.^28^ Scott et al. demonstrated that the CONCEPTT participants using CGM had significantly lower glucose (0.4–0.8 mmol/L [7–14 mg/dL]) for ∼7 h/day, mainly during the daytime (08.00–12.00 and 16.00–19.00) compared with women using SMBG.^28^ At 24 weeks of gestation, women using insulin pumps had similarly higher glucose levels for 12 h/day, during the entire afternoon (13.00–18.00 h), after dinner (20.30–00.30), and overnight (03.00–06.00 h) periods compared with those on multiple daily injection therapy. This suggests that women using multiple daily injections were able to more effectively match their prandial insulin dose calculations and the timing of their premeal boluses to their dietary intake, especially for their lunch and evening meals during the second trimester. The expected benefits of insulin pump therapy especially for overnight glycemia (03.00–06.00 h) were not apparent during midpregnancy. Importantly, these differences in glycemia between women using insulin pumps and multiple daily injections were not attributed to differences in maternal dietary intake.^29^

## The Use of CGM Is Only Part of Improving Outcomes in Type 1 and 2 Diabetes Pregnancies

The achievement of glucose targets in pregnancy is more common in type 2 diabetes compared with type 1 diabetes pregnancies.^5^ Despite this, there are emerging data from the United Kingdom and Scotland suggesting that the risk of serious adverse pregnancy outcomes (congenital anomaly, stillbirth, and neonatal death) is now even higher in type 2 than in type 1 diabetes pregnancy.^6,30^ Nationwide population-based data from Scotland and the United Kingdom both suggest higher perinatal death rates in type 2 compared with type 1 diabetes pregnancy.^6,30^ Maternal glucose was the dominant risk factor for perinatal death in both cohorts. It is well established that pregnant women with type 2 diabetes are older, have higher BMI, higher levels of social deprivation, and are more likely to belong to the black or Asian ethnic groups than pregnant women with type 1 diabetes.^31^ In addition to these complex demographic factors, younger women with type 2 diabetes also face substantial health care inequalities, with only a minority being offered and/or attending specialist prepregnancy clinics.^32,33^ As a consequence, despite their increased obstetric risk factors and added comorbidities of hypertension and hyperlipidemia, women with type 2 diabetes more frequently conceive while taking potentially harmful medications, without adequate folic acid supplementation and without insulin to optimize preconception glucose levels.^32,33^

In the UK nationwide population-based study, fewer than one in five women of reproductive years with type 2 diabetes were taking insulin at their first antenatal visit.^6^ While their HbA1c levels are (∼1% or 10 mmol/mol) lower compared with women with type 1 diabetes, still only one-third of women with type 2 diabetes achieve target HbA1c levels (<48 mmol/mol or 6.5%) in early pregnancy.^5^ Having type 2 diabetes for 5 years or longer, an overweight (BMI >25 kg/m^2^) or obese BMI category (BMI >30 kg/m^2^) that includes 90% of women entering pregnancy, higher deprivation, and black or Asian ethnicity are all associated with higher HbA1c levels (Table 1).^6^

As in type 1 diabetes, there was improvement across gestation but no significant clinic to clinic variation in glycemic attainment, either in early or late pregnancy. Thus, new interventions to optimize maternal glucose levels and reduce the modifiable risks for perinatal deaths are also urgently needed, for all pregnant women with type 2 diabetes, and across all maternity clinics.

The benefits of prepregnancy care for improving pregnancy preparation and reducing the risks of serious adverse pregnancy outcomes are as applicable to women with type 2 as those with type 1 diabetes.^32,33^ Likewise, the reductions in preterm births, large-for-gestational-age babies, and neonatal intensive care unit admissions associated with optimal second and third trimester glucose levels are also equally as applicable for pregnant women with type 2 diabetes. However, the stringent demands of monthly prepregnancy care clinics and the additional time and effort required to achieve and maintain optimal glucose levels may not be considered a high priority for disadvantaged women.^34^ Qualitative research suggests that we may also need to address the unconscious biases of women and health care professionals in relation to fertility in women of higher BMI and older maternal age.^35^

Rather than treating all pregnant women with type 1 and type 2 diabetes similarly, specifically targeted interventions according to the type of diabetes may be more applicable for addressing women's needs, improving pregnancy preparation, and reducing adverse pregnancy outcomes. While there is an urgent need to focus on supporting all women with diabetes to achieve a healthy BMI before pregnancy, we may need to strengthen the emphasis on maternal bodyweight and limiting gestational weight gain in type 1 diabetes and on optimizing maternal glucose levels in type 2 diabetes pregnancy.

## The Importance of Continued Care in the COVID-19 Pandemic

As a result of the pandemic, virtual care is becoming central to the management of diabetes in pregnancy. In some ways this may be more convenient for individuals with diabetes given the frequent visits required for insulin titration throughout pregnancy due to the dynamic changes in glucose metabolism and insulin resistance as pregnancy progresses. The role of online CGM and insulin pump platforms such as Clarity, CareLink, Diasend, and LibreView may be considerable during the pandemic as they allow individuals with diabetes teams to view CGM profiles remotely. There is also a variety of online resources to support diabetes self-management using technology during pregnancy (Table 2). The ability to view CGM profiles on a virtual platform can allow clinicians to offer individualized guidance to people with diabetes without the need for an in-person visit.

## Conclusions

CGM has transformed diabetes care in pregnancy with an increasing body of evidence demonstrating that CGM can improve maternal antenatal glucose levels and neonatal outcomes. Over time, CGM has become increasingly user-friendly and we now have glucose sensors that are approved for use during pregnancy. Online CGM platforms have given diabetes care providers access to CGM profiles in this increasing world of virtual care. While the use of CGM has been shown to improve pregnancy outcomes in type 1 diabetes, more data are needed in pregnancies complicated by gestational and type 2 diabetes. It is also clear that diabetes technology, whether it is CGM, insulin pump therapy, or closed-loop systems, cannot overcome all of the physiological and pharmacological challenges of pregnancy. Health care teams must continue to optimize the basics of diabetes treatment, including increasing access to contraception and prepregnancy care, timing and escalating prandial insulin dosing appropriate to advancing gestational age, and strategically utilizing dietary intake and daily exercise for optimal glycemia.
